# A randomized controlled trial of ivabradine in patients with acute myocardial infarction related cardiogenic shock

**DOI:** 10.47487/apcyccv.v5i2.342

**Published:** 2024-06-24

**Authors:** Alejandro Alcaraz-Guzmán, Eder Jonathan Amaro-Palomo, Arturo Maximiliano Ruiz-Beltrán, Braiana Ángeles Díaz-Herrera, Raúl Rodrigo Neri-Bale, Lilia Hernández-Bravo, Manuel A. Candia-Ramírez, Rodrigo Gopar-Nieto, Héctor González-Pacheco, Jorge Daniel Sierra-Lara Martinez, Alexandra Arias-Mendoza, Diego Araiza-Garaygordobil

**Affiliations:** 1 Coronary Care Unit, National Institute of Cardiology “Ignacio Chávez”, Mexico City, Mexico. Coronary Care Unit National Institute of Cardiology “Ignacio Chávez” Mexico City Mexico

**Keywords:** Ivabradine, Acute Myocardial Infarction, Cardiogenic Shock, Tachycardia, Cardiac Output, Pulmonary Artery Catheterization, Ivabradina, Infarto Agudo del Miocardio, Choque Cardiogénico, Taquicardia, Gasto Cardiaco, Cateterismo de la Arteria Pulmonar

## Abstract

**Objective:**

. Acute myocardial infarction-related cardiogenic shock (AMI-CS) is often accompanied by tachycardia, which, in turn, increases myocardial oxygen consumption and hinders the use of ventricular assist devices, such as intra-aortic balloon pump. Evidence suggests that ivabradine may reduce heart rate (HR) without affecting other hemodynamic parameters. The aim of the present study was to determine the effect of ivabradine on reducing HR and changes in other hemodynamic parameters such as cardiac index (CI), in patients with AMI-CS and tachycardia.

**Materials and methods:**

. A single-center, open label, randomized clinical trial included patients diagnosed with AMI-CS and tachycardia with >100 beats per minute (BPM). Heart rate, cardiac index, and other hemodynamic parameters measured by pulmonary flotation catheter were compared at 0, 6, 12, 24, and 48 hours after randomization.

**Results:**

. A total of 12 patients were randomized; 6 received standard therapy, and 6 received ivabradine in addition to standard therapy. Baseline clinical characteristics were similar at randomization. A statistically significant lower heart rate was found at 12 hours (p=0.003) and 48 hours (p=0.029) after randomization, with differences of -23.3 (-8.2 to -38.4) BPM and -12.6 (-0.5 to -25.9) BPM, respectively. No differences in cardiac index, or any other evaluated hemodynamic parameters, length of hospital stay, nor mortality rate were noted between both groups.

**Conclusions:**

. The use of ivabradine in patients with AMI-CS was associated with a significant reduction in heart rate at 12 and 48 h, without affecting other hemodynamic parameters.

## RESUMEN

**Objetivo:**

. El choque cardiogénico relacionado con el infarto agudo de miocardio (AMI-CS, por sus siglas en inglés) suele ir acompañado de taquicardia, lo que, a su vez, aumenta el consumo de oxígeno miocárdico y dificulta el uso de dispositivos de asistencia ventricular, como la bomba de balón intraaórtico. La evidencia sugiere que la ivabradina puede reducir la frecuencia cardíaca (FC) sin afectar otros parámetros hemodinámicos. El objetivo del presente estudio fue determinar el efecto de la ivabradina en la reducción de la FC y los cambios en otros parámetros hemodinámicos como el índice cardíaco (CI) en pacientes con AMI-CS y taquicardia.

**Materiales y métodos.:**

Se incluyeron pacientes diagnosticados con AMI-CS y taquicardia con >100 latidos por minuto (LPM) en un ensayo clínico aleatorizado de un solo centro. La frecuencia cardíaca, el índice cardíaco y otros parámetros hemodinámicos medidos mediante catéter de flotación pulmonar se compararon a las 0, 6, 12, 24 y 48 h después de la aleatorización.

**Resultados.:**

Se aleatorizaron un total de 12 pacientes; 6 recibieron terapia estándar y 6 recibieron ivabradina además de la terapia estándar. Las características clínicas basales fueron similares en la aleatorización. Se encontró una frecuencia cardíaca significativamente más baja a las 12 h (p=0,003) y a las 48 h (p=0,029) después de la aleatorización, con diferencias de -23,3 (-8,2 a -38,4) LPM y -12,6 (-0,5 a -25,9) LPM, respectivamente. No se observaron diferencias en el índice cardíaco, en ningún otro parámetro hemodinámico evaluado; tampoco en la duración de la estancia hospitalaria, ni en la tasa de mortalidad entre ambos grupos.

**Conclusiones.:**

El uso de ivabradina en pacientes con AMI-CS se asoció con una reducción significativa en la frecuencia cardíaca a las 12 y 48 h, sin afectar otros parámetros hemodinámicos.

## Introduction

Cardiogenic shock (CS) is defined as a critical state of organic hypoperfusion secondary to primarily reduced cardiac output due to cardiac dysfunction, which, if not reversed, culminates in multiorgan failure and death ^1^. CS is often accompanied by tachycardia, which has a compensatory nature. Furthermore, inotropic and/or vasopressor therapy administered to restore tissue perfusion frequently leads to tachycardia. However, an inappropriate increase in heart rate (HR) reduces the diastolic period and systolic volume, which can be detrimental, as it increases myocardial oxygen consumption and reduces coronary perfusion ^1^^-^^3^.

The hyperpolarization-activated cyclic nucleotide-gated (HCN) transmembrane channels are encoded by a family of four genes (HCN1-4). These channels are prominently expressed in the heart, with HCN4 being the most abundant in the sinoatrial node, where it plays a pivotal role in initiating the cardiac cycle through the generation of the “funny” current (If), which triggers depolarization in the sinoatrial node ^4^^-^^6^.

Ivabradine, a selective inhibitor of HCN4, reduces heart rate through a decrease in the diastolic depolarization slope of the pacemaker action potential ^7^^-^^9^. Ivabradine has been studied as an anti-anginal medication and as a treatment for patients with heart failure with reduced ejection fraction (HFrEF) to reduce hospitalizations and mortality ^10^^-^^13^. However, the potential to reduce HR in patients with AMI-CS and tachycardia has not been prospectively addressed in a randomized controlled trial.

The aim of the present study was to determine the effect of ivabradine on reducing HR and changes in other hemodynamic parameters such as cardiac index (CI) in patients with AMI-CS and tachycardia.

## Materials and methods

### Study design

We designed and conducted a randomized, controlled, open-label clinical trial including all adult patients with AMI-CS and tachycardia who were hospitalized in the coronary care unit of the study center and in whom there was willingness and agreement of the treating physician to use ivabradine (off-label [used for a purpose other than that for which it has been officially approved by regulatory authorities] in our country where the study was conducted) in the context of AMI-CS.

The patients were randomized using a 2x2 permuted block design facilitated by the Randomization.com platform. The corresponding author generated the randomization sequence. Patient enrollment and assignment to study groups were carried out by the principal investigator under the supervision of the corresponding author. They were assigned to receive either standard treatment alone or standard therapy plus ivabradine at doses of 5mg orally administered twice daily to conscious patients. For patients unable to swallow, such as those undergoing invasive mechanical ventilation, ivabradine was administered via an orogastric tube ^5^.

### Population 

AMI-CS was defined as a systolic blood pressure of less than 90 mmHg for 30 minutes, despite adequate volume resuscitation, or the need for vasopressor +/- inotropic therapy to maintain blood pressure >90 mmHg, in addition to hypoperfusion (either lactate >2.0 mmoL/L or an hourly diuresis of less <0.5 ml/kg) and pulmonary congestion (either crackles, congestion in chest X-ray or B-lines in lung US) ^1^^,^^13^. Furthermore, in line with the methodology outlined in the SHOCK trial, which utilized a cardiac index (CI) of ≤2.2 L/min per m2 and a pulmonary capillary wedge pressure (PCWP) of ≥15 mm Hg for the diagnosis of AMI-CS, we adopted a similar approach with the assistance of the pulmonary flotation catheter ^14^.

Acute MI was defined following the fourth universal definition as the presence of acute myocardial injury detected by abnormal cardiac biomarkers in the setting of clinical symptoms, electrocardiographic changes, or imaging evidence (such as echocardiography) ^15^. Tachycardia was defined as a mean HR of >100 beats per minute (BPM) for at least 3 hours (according to continuous patient HR monitoring) ^16^.

The exclusion criteria were patients with non-sinus rhythm (ie: atrial fibrillation), the presence of a sinoatrial or atrioventricular block of any degree at the time of diagnosis, and a history of ivabradine intolerance or side effects. The elimination criteria were death within the first 24 hours of hospital admission, removal of the pulmonary flotation catheter before 24 hours after initiation of treatment, and development of bradycardia less than 40 BPM at any time after randomization.

### Data collection

Hemodynamic parameters at 0, 6, 12, 24, and 48 hours were determined using a pulmonary flotation catheter placed through internal jugular vein: HR, CI, mean arterial pressure (MAP), mean pulmonary arterial pressure (MPAP), pulmonary artery occlusion pressure (PAOP), central venous pressure (CVP), indexed stroke volume (SVI), indexed systemic vascular resistance (SVRI), cardiac output (CO) and cardiac power (CP). Additionally, the time required for withdrawal of inotropic support, withdrawal of vasopressor support, withdrawal of mechanical circulatory support, length of hospital stay, and survival rate were also evaluated.

### Sample size

Considering a statistical power of 90% (beta-1), a two-tailed significance level (alpha) of 0.05, and based data from Chiu, *et al*.^17^ where a reduction in HR of 14.4 BPM with the use of ivabradine in CS was demonstrated at 24 hours after the start of treatment, five patients per group were required to demonstrate statistically significant differences in HR. Considering possible losses (20%), six patients per group (a total of 12) were included ^17^.

### Statistical analysis

For the description of baseline characteristics, binary variables were described as frequencies and proportions and analyzed using Pearson’s chi-squared test (χ²) or Fisher’s exact test, depending on the number of individuals. Quantitative variables were analyzed for normality using the Shapiro-Wilk’s test and described as parametric or non-parametric accordingly. Conforming to the results of distribution tests, we used either the T-test or the Wilcoxon-Mann-Whitney rank sum test.

We employed the Wilcoxon-Mann Whitney test for non-parametric data and the T-test for normally distributed data to compare differences between groups. For assessing the primary objective, we applied a t-test for independent samples, as well as t-test for paired samples. Significance was established at the conventional threshold of p≤0.05 for both tests. Our statistical analysis was conducted using STATA v14.1 (StataCorp LP, College Station, TX) for its capability to manage complex datasets and conduct comprehensive statistical analyses.

### Ethics approval and consent to participate

The present protocol was approved by the Ethics and Research committee from the Education Department at the author’s Institution. Data on patients was collected in accordance with the 1975 guidelines of the Declaration of Helsinki. Informed consent was given prior to randomization, at the time participants met the inclusion criteria described in our study.

It is important to note that in all patients randomized to our study, the pulmonary flotation catheter (Swan-Ganz) had been previously placed due to the patient’s clinical condition, as this catheter is used in approximately 60% of patients diagnosed with cardiogenic shock and acute myocardial infarction in our center. However, if the patient was not in adequate neurological or reasoning conditions, such as in the case of the patient under invasive mechanical ventilation, informed consent was offered to two responsible family members of the patient, who authorized and agreed to their randomization into the trial.

## Results

### Baseline characteristics

**Figure 1:**
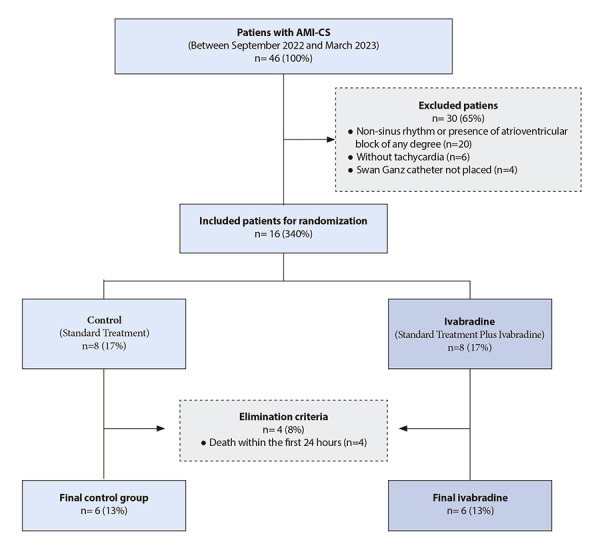
Flow chart for study participants.

Between September 2022 and March 2023, 12 patients were included, six patients received ivabradine orally plus standard therapy, and six patients received standard treatment alone (Figure 1).

All patients recruited for the study had STEMI (ST-elevation myocardial infarction), and all of them were men with an average age of 60.2 ± 9.2 years. Patients in the ivabradine group were older (65 vs. 54 years, p=0.027). Time from hospital admission to randomization was similar in both groups (ivabradine: 31.5 hours [IQR: 24.5-57] vs. control: 36.5 hours [IQR: 27-42]) and the rest of baseline clinical characteristics were also similar among groups (Table 1).

**Table 1:** Baseline characteristics according to randomization

	Total	Ivabradine	Control	p
Age, mean (±SD), years	60.2 (9.2)	65.8 (5.7)	54.6 (8.8)	0.027*
Men, n (%)	12 (100%)	6 (100%)	6 (100%)	1.000
BSA, median (IQR), m^2^	1.78 (1.69-1.83)	1.71 (1.68-1.78)	1.82 (1.79-1.84)	0.529
BMI, media (IQR), kg/m^2^	27.6 (3.7)	28.4 (4.1)	26.8 (3.5)	0.502
Hypertension, n (%)	7 (58.3%)	3 (50%)	4 (66.6%)	0.558
DM, n (%)	8 (66.6%)	4 (66.6%)	4 (66.6%)	1.000
Smoking, n (%)	10 (83.3%)	4 (66.6%)	6 (100%)	0.121
Previous MI, n (%)	2 (16.6%)	1 (16.6%)	1 (16.6%)	1.000
Previous PTCA, n (%)	1 (8.3%)	0 (0%)	1 (16.6%)	0.296
Reperfusion, n (%)	9 (75%)	5 (83.3%)	4 (66.6%)	0.505
PTCA	5 (41.6%)	3 (50%)	2 (33.3%)	
Thrombolysis	4 (33.3%)	2 (33.3%)	2 (33.3%)	
Location of MI, n (%)				
Inferior	3 (25%)	1 (16.6%)	2 (33.3%)	0.164
Anterior	7 (58.3%)	5 (83.3%)	2 (33.3%)	
Lateral	2 (16.5%)	0 (0%)	2 (33.3%)	
Responsible artery for infarction, n (%)				
RCA	2 (16.6%)	1 (16.6%)	1 (16.6%)	0.117
LAD	7 (58.3%)	5 (83.3%)	2 (33.3%)	
Circumflex	3 (25.0%)	0 (0%)	3 (50%)	
LVEF, median (IQR), %	20.5 (18.5-30)	24 (17-33)	20 (20-24)	0.532
Hs-cTnT, median (IQR), ng/ml	8692 (1645.5-35291.5)	35291.5 (1760-49723)	3165 (1531-14145)	0.062
NT-proBNP, median (IQR), pg/ml	4893.5 (2534-7867)	4157 (1420-7437)	5288 (4562-8297)	0.887
STEMI, n (%)	12 (100%)	6 (100%)	6 (100%)	1.000
SCAI Classification, n (%)				
C	3 (25%)	1 (16.6%)	2 (33.3%)	
D	7 (58.3%)	4 (66.6%)	3 (50%)	0.78
E	2 (16.6%)	1 (16.6%)	1 (16.3%)	0.78
Use of inotropic support, n (%)	11 (91.6%)	6 (100%)	5 (83.3%)	0.296
Use of vasopressor support, n (%)	8 (66.6%)	5 (83.3%)	3 (50%)	0.221
Use of mechanical circulatory support, n (%)	7 (58.3%)	3 (50%)	4 (66.6%)	
IABP	6 (50%)	2 (33.3%)	4 (66.6%)	0.558
Impella	1 (8.3%)	1 (16.6%)	0 (0%)	
Use of invasive mechanical ventilation, n (%)	4 (33.3%)	3 (50%)	1 (16.6%)	0.194
Hospital admission to randomization time, median (IQR), hours	33.5 (24.5-57)	31.5 (22-72)	36.5 (27-42)	0.68

BMI=Body mass index. BSA=Body surface area. DM=Diabetes mellitus. HF=Heart failure. Hs-cTnT=High sensitivity cardiac troponin T. IABP=Intra-aortic balloon pump. LAD=Left anterior descending. LVEF=Left Ventricular ejection fraction. MI=Myocardial infarction. NT-proBNP=N-terminal pro brain natriuretic peptide. PTCA=Percutaneous transluminal coronary angioplasty. RCA=Right coronary artery. STEMI=ST-segment elevation myocardial infarction.

* Statistically significant difference

### Hemodynamic and clinical outcomes

**Figure 2:**
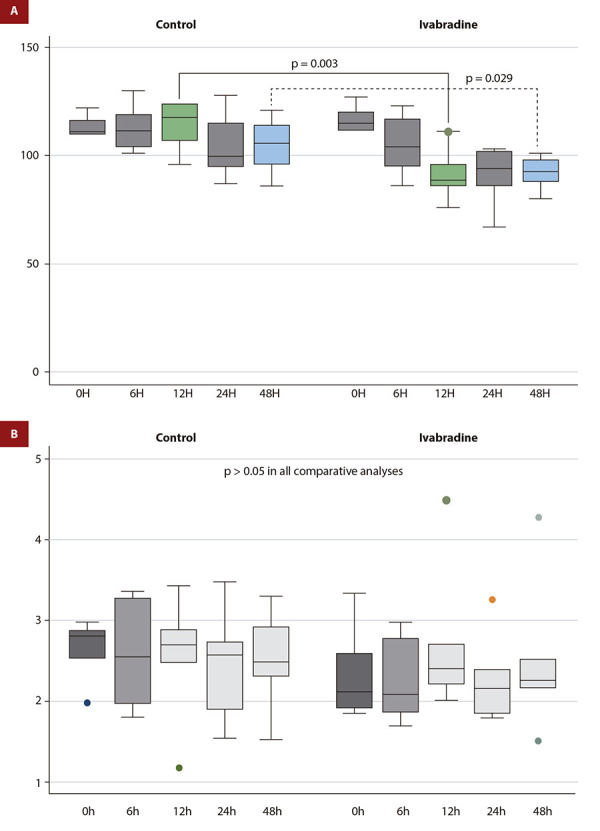
(A) Heart rate (beats per minute) at different time points (0h, 6h, 12h, 24h, 48h) according to the treat ment (ivabradine or control). Significant p-values are mentioned; the remaining comparisons demonstrated a p-value >0.05. (B) Cardiac index (L/min/m2) at different time points (0h, 6h, 12h, 24h, 48h) according to the treatment (ivabradine or control). All comparisons with a p-value > 0.05.

**Figure 3:**
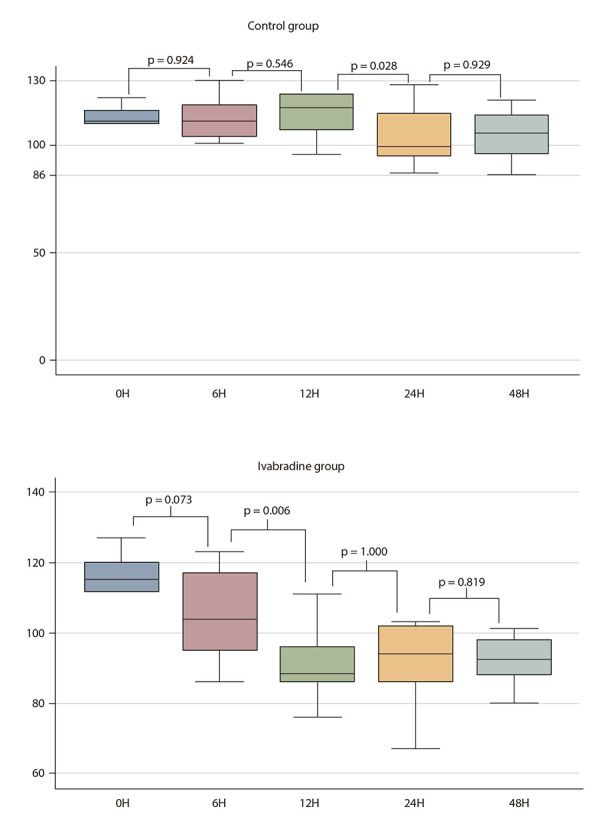
Figure illustrating the results of paired comparisons for heart rate in control & ivabradine arms.

A statistically significant lower HR was found at 12 and 48 hours in patients randomized to ivabradine, with a difference of -23.3 (-8.2 to -38.4, p=0.003) BPM and -12.6 (-0.5 to -25.9, p=0.029) BPM, respectively (Figure 2, panel A). While mean HR was not different among groups at 24 and 48 h, a paired-sample test assessment demonstrated a statistically significant reduction in HR (compared to baseline) at 24 h (-25.8 [-14.2 to -34.7] BPM, p=0.001) and 48 h (-24.8 [-17.6 to -31.9] BPM, p=0.0001) in patients randomized to ivabradine, but not in patients randomized to standard therapy (24h: -9.3 [-25.5 to +6.8] BPM, p=0.09; 48 h: -8.6 [-23.0 to +5.7] BPM, p=0.09); (Figure 3).

CI was not different at any time point (Figure 2, panel B). There were no significant differences between the treatment groups in any hemodynamic parameters at 6, 12, 24, and 48 hours (Table 2), nor were there any significant differences in the use of inotropic or vasopressor medications or the use of mechanical circulatory support devices between the groups.

**Table 2:** Hemodynamic outcomes

	Baseline	6 hours	12 hours	24 hours	48 hours
HR, bpm					
Total	115.0 (5.4)	108.8 (12.7)	102.6 (16.5)	97.5 (15.2)	98.3 (11.8)
Ivabradine	116.8 (5.8)	104.8 (13.8)	91.0 (11.7)	91.0 (13.6)	92.0 (7.4)
Control	113.3 (4.8)	112.8 (11.3)	114.3 (11.6)	104.0 (15.0)	104.6 (12.5)
Difference	3.5	-8	-23.3	-13	-12.6
*p* value	0.859	0.149	0.003*	0.074	0.029*
MAP, mmHg					
Total	82.5 (11.5)	79.9 (10.8)	77.5 (12.8)	74.8 (8.0)	77.4 (9.0)
Ivabradine	78.6 (12.5)	79.3 (7.3)	78.1 (13.8)	74.0 (8.7)	74.6 (9.8)
Control	86.3 (10.0)	80.5 (14.2)	77.0 (13.0)	75.6 (8.0)	80.1 (8.0)
Difference	-7.6	-1.1	1.1	-1.6	-5.5
*p* value	0.134	0.431	0.558	0.369	0.156
MPAP, mmHg					
Total	27.4 (8.9)	26.5 (5.4)	25.0 (2.2)	24.4 (4.3)	25.5 (7.5)
Ivabradine	28.0 (10.6)	24.5 (4.5)	22.8 (4.0)	23.1 (3.3)	22.8 (6.0)
Control	26.8 (7.9)	28.6 (5.9)	27.1 (10.0)	25.6 (5.2)	28.2 (8.4)
Difference	1.1	-4.1	-4.3	-2.5	-5.4
*p* value	0.582	0.100	0.175	0.172	0.139
PAOP, mmHg					
Total	15.8 (5.3)	13.6 (4.2)	15.6 (5.9)	15.0 (3.8)	13.6 (4.0)
Ivabradine	15.1 (5.8)	12.8 (4.8)	15.1 (7.3)	14.3 (3.8)	12.4 (5.0)
Control	16.5 (5.2)	14.5 (3.9)	16.1 (4.8)	15.8 (4.1)	14.8 (2.5)
Difference	-1.3	-1.6	-1	-1.5	-2.4
*p* value	0.343	0.263	0.393	0.265	0.187
CVP, mmHg					
Total	10.5 (3.7)	10 (5.0)	8.9 (5.3)	8.7 (3.8)	11.1 (5.7)
Ivabradine	11.6 (3.9)	11.1 (6.8)	9.1 (6.3)	9.1 (4.4)	9.6 (7.0)
Control	9.5 (3.6)	8.8 (2.3)	8.6 (4.8)	8.3 (3.6)	12.6 (4.3)
Difference	2.1	2.3	0.5	0.8	-3.0
*p* value	0.827	0.776	0.559	0.635	0.220
SVI, ml/m^2^					
Total	19.4 (5.7)	22.2 (8.6)	22.6 (10.1)	20.3 (7.2)	21.4 (7.1)
Ivabradine	17.7 (5.7)	22.8 (7.2)	26.0 (11.7)	20.4 (5.0)	22.4 (4.0)
Control	21.1 (5.6)	21.6 (10.5)	19.2 (7.8)	20.2 (9.5)	20.3 (5.5)
Difference	-3.4	1.2	6.8	0.2	2.1
p value	0.161	0.592	0.868	0.520	0.667
SVRI, DSm^2^ /cm^5^					
Total	2473 (595)	2384 (441)	2208 (532)	2331 (542)	2235 (634)
Ivabradine	2351 (296)	2516 (524)	2149 (453)	2369 (564)	2216 (691)
Control	2596 (810)	2251 (333)	2267 (640)	2293 (570)	2255 (652)
Difference	-245	264	-117	76	-38
*p* value	0.936	0.336	0.521	0.748	0.916
CO, L/min					
Total	4.31 (0.98)	4.30 (0.95)	4.69 (1.41)	4.21 (0.94)	4.50 (1.30)
Ivabradine	4.13 (1.04)	3.98 (0.85)	4.82 (1.63)	4.05 (1.01)	4.55 (1.71)
Control	4.48 (0.97)	4.62 (1.00)	4.57 (1.30)	4.38 (0.94)	4.45 (0.95)
Difference	-0.34	-0.64	0.24	-0.33	0.10
p value	0.284	0.128	0.610	0.283	0.544
CI, L/min/m^2^					
Total	2.49 (0.48)	2.41 (0.58)	2.63 (0.80)	2.36 (0.59)	2.52 (0.82)
Ivabradine	2.32 (0.56)	2.24 (0.52)	2.70 (0.91)	2.46 (0.67)	2.54 (1.03)
Control	2.66 (0.36)	2.58 (0.64)	2.56 (0.75)	2.26 (0.53)	2.50 (0.67)
Difference	-0.34	-0.34	0.14	0.19	0.038
*p* value	0.120	0.170	0.613	0.294	0.526
CP, Watts					
Total	0.79 (0.24)	0.76 (0.24)	0.82 (0.33)	0.69 (0.18)	0.76 (0.24)
Ivabradine	0.73 (0.28)	0.69 (0.16)	0.84 (0.34)	0.65 (0.17)	0.74 (0.29)
Control	0.85 (0.20)	0.83 (0.31)	0.80 (0.34)	0.73 (0.20)	0.78 (0.22)
Difference	-0.11	-0.14	0.04	-0.08	-0.04
*p* value	0.223	0.172	0.583	0.224	0.398

CI=Cardiac Index. CP=Cardiac power. CO=Cardiac output. CVP=Central venous pressure. SVI=Indexed stroke volume. SVRI=Indexed systemic vascular resistance. HR=Heart Rate. MAP=Mean arterial pressure. MPAP=Mean pulmonary arterial pressure. PAOP=Pulmonary artery occlusion pressure.

* Statistically significative difference

Values expressed as mean (±SD)

Finally, there were no significant differences in the length of hospital stay or the survival rate between groups (Table 3). The average length of hospital stay was 13 ± 5 days. Ten patients were discharged alive from the hospital, while two patients from the ivabradine group died during hospitalization; in both cases, infectious complications that led to sepsis and septic shock were attributed as the cause of death.

**Table 3:** Clinical outcomes and support therapy during the study

	Total	Ivabradine	Control	p value
Length of Hospital Stay, days	13.9 (5.2)	13.6 (6.3)	14.1 (5.2)	0.809
Survival, n (%)	10 (83.3%)	4 (66.6%)	6 (100%)	0.121
Duration of inotropic support, hours	101 (34.2)	110 (34.1)	91 (39.1)	0.273
Duration of vasopressor support, hours	54.8 (41.5)	46.2 (40.0)	69.3 (56.7)	0.456
Duration of mechanical circulatory support, hours	72 (26.9)	94.6 (26.7)	55 (17.7)	0.077

Values expressed as mean (±SD), except survival (n and %)

## Discussion

The present study suggests that in patients with AMI-CS and tachycardia, ivabradine is associated with a lower HR without affecting other hemodynamically significant parameters (such as CI). These results may be of interest to treat those patients in which tachycardia may be deleterious or may impede other therapeutic maneuvers (such as IABP inflation/deflation). Despite most patients receiving vasopressor and/or inotropic support, as well as mechanical circulatory support that could modify hemodynamics parameters, there were no significant differences between both groups, as well as the time of their hospital admission and randomization, which also did not demonstrate significance.

Furthermore, given the critical clinical condition of patients upon admission, and considering the period elapsed from admission to randomization during which they received assistance with inotropic/vasopressor agents or mechanical circulatory support, this observation may elucidate the achievement of target values for cardiac index and central venous pressure (CVP) in the measured parametric data.

The discrepancy observed in heart rate between the 12 and 48-hour intervals exhibited a statistically significant difference, while the heart rate at 24 hours did not reach the p-value threshold. Nevertheless, these findings approached statistical significance, suggesting that the observed trend may have been constrained by the limited sample size, thereby compromising statistical power.

These findings, observed at 12 hours and 48 hours but not at 24 hours, could be consistent with the pharmacodynamic effect of ivabradine, which has a distribution half-life of two hours and an effective half-life of approximately six hours. However, this presents a situation where the heart rate does not decrease further despite increasing the dosage of ivabradine beyond a certain point. This means that after reaching a certain dosage level, the effect of ivabradine on reducing heart rate stabilizes or levels off instead of continuing to decrease linearly ^18^.

Our results are consistent with those reported by Chiu *et al.*^17^, who found a significant reduction in HR (91.6 ± 6.4 vs. 106 ± 6.8 BPM, p=0.04) 24 hours after starting ivabradine in 5 patients with CS (4 patients with non-ischemic CS) in a non-randomized study. Patients receiving ivabradine also demonstrated an increase in stroke volume and RV and LV stroke work index without significant changes in MAP or CI. 

Similarly, Pascual *et al.*^11^ found an absolute reduction of 10 BPM at 6 h (p<0.001), 11 BPM at 24 h (p=0.004), and 19 BPM (p<0.001) at discharge after the initiation of ivabradine in 29 patients with acute HF and catecholamine-induced tachycardia, in a retrospective, non-randomized study. No episodes of hypotension or bradycardia were seen, and the authors hypothesize that ivabradine may be of use in this population. 

While our study demonstrated a statistically significant reduction in HR with the use of ivabradine, in contrast to findings in prior research ^19^^-^^21^, our study observed no differences (either positive or negative) in other hemodynamically relevant parameters (such as CI or PAOP) or other clinically meaningful parameters such as the time required for the withdrawal of life support or length of hospital stay. No adverse effects related to ivabradine, such as bradycardia, were observed in the patients of this group.

It is important to note that during the analysis of our study, two patients from the ivabradine group passed away, as described in **Table 3**. However, this analysis does not specifically outline the mortality percentage, rather, it only accounts for the patients who died during our trial. It is pertinent to emphasize that CS-AMI is a condition associated with high mortality, so when combining the two patients who passed away and were followed up, along with the four patients who were excluded due to early demise, aligns with mortality rates reported in international studies ^22^. Finally, there was no significant difference in mortality observed between the control group and the ivabradine group.

The major limitation of our study is the sample size, clearly underpowered to detect meaningful differences in clinical endpoints. Other limitations include the open-label nature and the exclusion of other causes of CS given that our study population is limited solely to patients with AMI-CS, and other causes of CS were not included. Finally, while the sample size was estimated to account for an alpha level of 0.05, the potential for type 1 error is still 5%, so further studies larger in sample size may be needed to confirm our results. 

In conclusion, we found that the use of ivabradine in patients with AMI-CS and tachycardia appears to be effective in reducing HR without deleteriously affecting other hemodynamic parameters in the short term. Future randomized clinical studies with a larger number of patients to evaluate the impact of ivabradine in clinical events in patients with AMI-CS are needed.
